# Effect of *Moringa oleifera* Lam. Leaf Tea on Sexual Behavior and Reproductive Function in Male Rats

**DOI:** 10.3390/plants10102019

**Published:** 2021-09-26

**Authors:** Jiraporn Laoung-on, Kanokporn Saenphet, Churdsak Jaikang, Paiwan Sudwan

**Affiliations:** 1 Department of Anatomy, Faculty of Medicine, Chiang Mai University, Chiang Mai 50200, Thailand; jiraporn_laoung@cmu.ac.th; 2 Department of Biology, Faculty of Science, Chiang Mai University, Chiang Mai 50200, Thailand; kanokporn.saenphet@cmu.ac.th; 3 Toxicology Section, Department of Forensic Medicine, Faculty of Medicine, Chiang Mai University, Chiang Mai 50200, Thailand; churdsak.j@cmu.ac.th

**Keywords:** *Moringa oleifera*, phytochemistry, sexual behavior, testicular function, male fertility, antioxidants

## Abstract

*Moringa oleifera* Lam. (*M*. *oleifera*) has been called the “miracle tree” due to its attributes for therapy in various diseases. However, *M*. *oleifera* studies on the male reproductive system have been limited. The aim of this study was a phytochemical screening and investigation of the effects of *M*. *oleifera* leaf tea on sexual behavior, reproductive function and antioxidant activities. Male rats were fed with *M*. *oleifera* leaf tea extract at doses of 0.55, 1.10 and 2.20 mg/kg or distilled water for 30 days. The result showed that *M*. *oleifera* leaf tea contained rich total phenols and flavonoids. The courtship behavior, seminiferous tubule diameter, epithelium height, epithelium area, type A spermatogonia, and spermatogonia efficiency were significantly increased in all treatment groups. The groups treated with 1.10 and 2.20 mg/kg increased the number of Sertoli cells. The total of spermatogenic cells increased in the group treated with 0.55 mg/kg. *M*. *oleifera* leaf tea scavenged DPPH radical, ABTS radical and H_2_O_2_, and inhibited LPO and AGEs formation. Due to *M*. *oleifera* leaf tea containing rich total phenols, flavonoids, and antioxidants, it could enhance sexual function and the male reproductive system.

## 1. Introduction

*Moringa oleifera* Lam. (*M*. *oleifera*) is a widely applied, studied, and cultivated species of the Moringaceae family [1]. This plant is reported as native to the Himalayan foothills of Northern India and is widely cultivated in the tropics and sub-tropics of Asia and Africa [2,3]. *M*. *oleifera* has been denominated the “miracle tree” due to its attributed positive impact on human health [4]. Ayurvedic traditional medicine reported that *M. oleifera* leaves can prevent and cure various diseases [5]. *M*. *oleifera* has been used as a nutrient supplement and to boost the immune system of HIV infected people in Zimbabwe [6]. Virtual screening and molecular dynamics stimulation of *M. oleifera* were performed to identify potential SARS-CoV-2 M^pro^ inhibitors and three flavonoids substances were identified, which may be developed as a potential protective and therapeutic agent against COVID-19 with a computer drug design method [7]. The traditional Siddha healers in the Virudhunagar district of Tamil Nadu, India used the *M*. *oleifera* flower as an aphrodisiac [8]. The aqueous extract of seed, fruit, and leaves of *M. oleifera* were found to contain vitamins, gallic acid, chlorogenic acid, ellagic acid, ferulic acid, kaempferol, quercetin, and vanillin [2]. Previous studies have reported that male rats treated with aqueous extract and ethanol extract of the *M. oleifera* seed had significantly increased sperm concentration [9,10], sperm motility, sperm viability and normal sperm morphology [10]. However, the male rats treated with the white seed powder of *M. oleifera* had significantly decreased sperm concentration, sperm motility and sperm morphology [11]. Regarding the previous experiment on the impact of *M. oleifera* on sperm characteristics, the results showed differences, which indicated that the different stage of the plant and the different methods of extraction can affect the sperm characteristics. In addition, the ethanolic extract of *M*. *oleifera* leaves improved sexual performance in stress-exposed rats and had aphrodisiac potential [12]. The leaves of *M*. *oleifera* were reported to have a greater potential inhibitory effect on lipid peroxidation (LPO), protein oxidation, and DNA damage than the fruits or seeds [2]. The leaves can be consumed in the form of food, medicine, and nutritional supplements [13]. They contain a strong natural antioxidant [14] and have been used in antidiabetic treatment [15,16], against oxidative stress [17,18], as an antimicrobial [17], and to prevent age-related diseases [2]. However, the method, the polarity of the solvent, the temperature, and the duration of extraction affect the contents of the bioactive component in the plant’s leaves [19,20]. The leaf stage reports showed that the mature leaves of *M*. *oleifera* had higher total phenol, total flavonoid, and antioxidant potential than tender leaves, indicating the mature leaf stage enhanced the human antioxidant protection system against oxidative damage [21]. Furthermore, environmental stress, such as light, temperature, soil nutrients and moisture stress affected the quality of phytomedicine in the plant extract [22]. The *M*. *oleifera* leaves contain rich phenols and flavonoids as natural antioxidants and can enhance male reproductive function [12].

Male reproductive dysfunction may be caused by poor lifestyle choices, certain occupations and age-related diseases, which will result in oxidative stress formation [23]. Oxidative stress causes lipid peroxidation, protein oxidation and alters DNA resulting in macromolecule damage in living cells [24,25,26], leading to a decrease in spermatogenesis, the physiological capacity of fertilization and male fertility [25]. The prevention and management of oxidative stress can improve male reproductive health. The main determinant of oxidative protection is the consumption of an antioxidant rich diet [25]. Plants rich in phytochemical compounds contain a good quantity of natural antioxidants and serve as an alternative choice for oxidative protection [24,25]. 

Although, *M*. *oleifera* leaves have previously been reported to enhance sexual activity, environmental stress, leaf stage, type of solvent, and method, temperature and duration of extraction affect the contents of bioactive components in the plant’s leaves [19,20,21,22]. Moreover, the aqueous extract from *M*. *oleifera* leaves, which is extracted using a method similar to how the Thai people prepare tea, to date has a little scientific research reported on its effects on male reproduction. Therefore, this study was developed using *M. oleifera* leaf tea from mature leaves and investigated the phytochemicals, antioxidant activity, and effects of *M. oleifera* leaf tea on sexual behavior and reproductive function in mature male rats. 

## 2. Results

### 2.1. Phytochemical Contents

**Figure 1:**
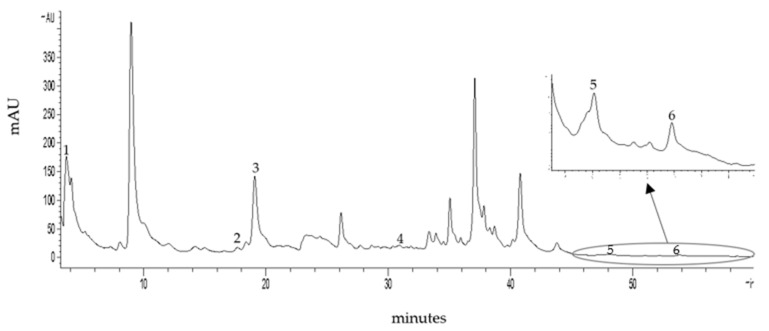
HPLC chromatograms of total phenols shown in the *M. oleifera* leaf tea from Column, Purospher^®^ Star PR-18; mobile phase, 0.1% formic acid in water and ACN; flow rate, 0.8 mL/min; detection wavelength, 250 nm. Peak identification: peak 1, gallic acid; peak 2, chlorogenic acid; peak 3, caffeic acid; peak 4, ferulic acid; peak 5, quercetin; peak 6, kaempferol.

Total phenolic, total flavonoid and total alkaloid contents in *M. oleifera* leaf tea showed 34.44 ± 0.99 µg gallic acid equivalent (GAE)/mg dried weight, 13.45 ± 3.03 µg quercetin (QE)/mg dried weight and 2.20 ± 0.01 ng morphine equivalent (ME)/mg dried weight, respectively. High-performance liquid chromatography (HPLC) screening for phenolics and flavonoids (250 nm) in the *M. oleifera* tea was adequately separated within 60 min (Figure 1). Retention time and standard UV spectrum of the phytochemical standard were used for identification. Gallic acid, chlorogenic acid, caffeic acid, ferulic acid, quercetin and kaempferol were found in the *M. oleifera* leaf tea and the retention times were 3.67, 17.66, 19.08, 31.85, 48.14 and 53.81 min, respectively. The amounts of gallic acid, chlorogenic acid, caffeic acid, ferulic acid, quercetin and kaempferol were 205.91, 48.80, 205.99, 8.53, 27.01 and 10.72 µg/mg dried weight, respectively.

### 2.2. Antioxidant Properties

**Figure 2:**
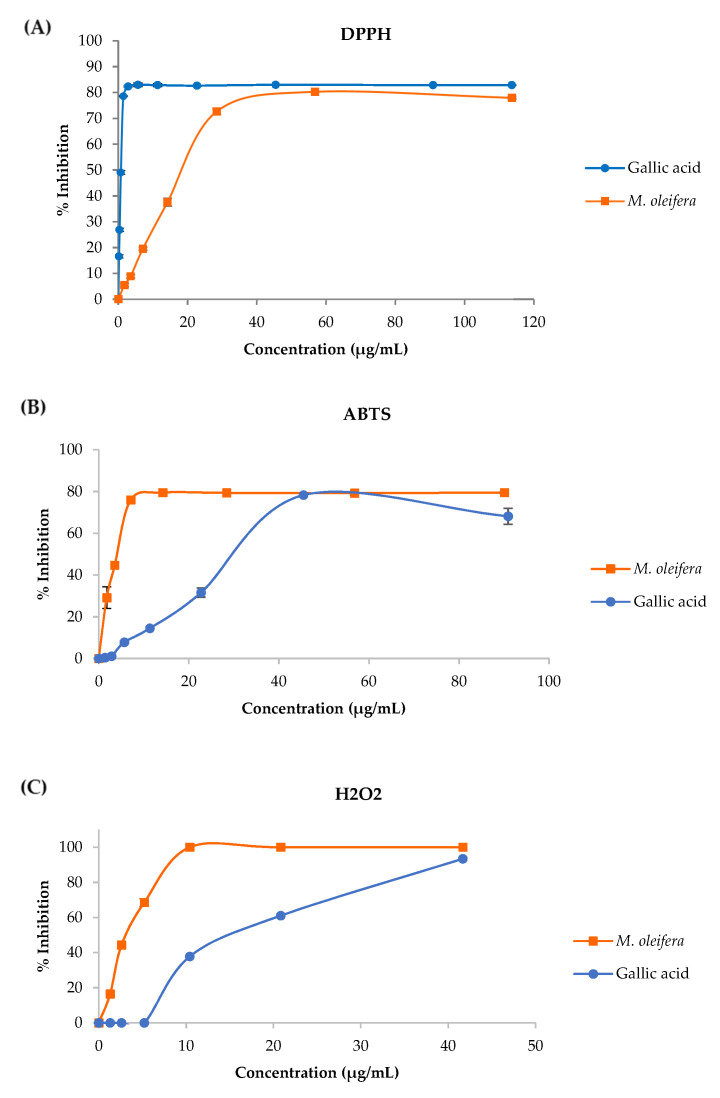
Percent inhibition of DPPH (**A**), ABTS (**B**), and H_2_O_2_ (**C**) of *M. oleifera* leaf tea. Data are mean values ± standard error (error bars).

The effects of *M. oleifera* leaf tea on 2,2-diphenyl-1-Picrylhydrazyl (DPPH), 2,2′-Azino-di- [3-Ethylbenzthiazoline Sulfonate] (ABTS) and hydrogen peroxide (H_2_O_2_) scavenging were investigated. The half-maximal inhibitory concentration (IC50) values of *M. oleifera* tea are presented in Table 1 and the *M. oleifera* leaf tea scavenged DPPH radical, ABTS radical, and H_2_O_2_ in a dose-dependent manner are shown in Figure 2A–C, respectively. The *M. oleifera* leaf tea had less potential DPPH radical scavenging than gallic acid (used as a standard). However, the *M. oleifera* leaf tea had greater potential ABTS radical scavenging and H_2_O_2_ radical scavenging compared to gallic acid.

### 2.3. Lipid Peroxidation (LPO), and Advance Glycation End Products (AGEs) Inhibition Assay

The *M. oleifera* leaf tea inhibited LPO and AGEs formation in in vitro models and the results are presented in Table 1. 

**Figure 3:**
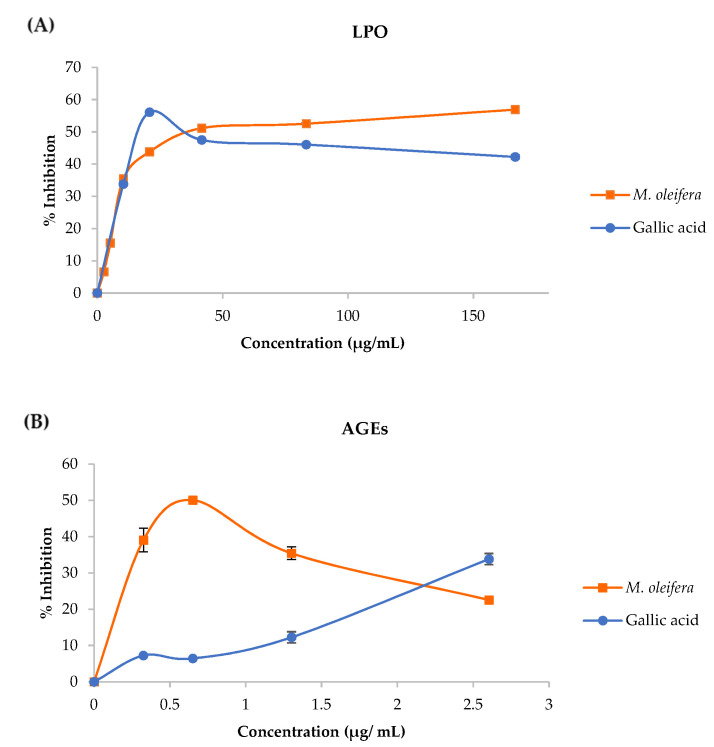
Percent inhibition of LPO (**A**) and AGEs (**B**) of *M. oleifera* leaf tea. Data are mean values ± standard error (error bars).

The inhibition of LPO with *M. oleifera* leaf tea was induced by FeSO_4_ in the linoleic acid model. The percent inhibitions shown in Figure 3A indicate that the leaf tea inhibited the LPO formation. The *M. oleifera* leaf tea inhibited AGEs formation, induced by D-glucose in the BSA model. The percent inhibitions are shown in Figure 3B, implying that the plant extracts inhibited the AGEs formation.

### 2.4. Sexual Behavior

All the treatment groups significantly increased courtship behavior (*p* < 0.05) within 30 min of observation compared with the control group. The mount latency (ML), intromission latency (IL), mount frequency (MF), intromission frequency (IF) and copulatory efficiency (CE) in all treated groups were similar to the control group (Table 2). However, the study of three 10-min intervals revealed that the control and treated groups of rats had significantly longer periods of courtship behavior displayed in the first 10-min interval than the second and third 10-min intervals of observations (Table 3). However, the data of MF and IF had a non-normal distribution therefore these parameters were analyzed using non-parametric analysis. There were no significant differences between MF and IF observed in the three intervals (Table 4 and Table 5). All treated groups had significantly increased courtship behavior in the first and second 10-min intervals of observation when compared to the control group but no significant difference in the third 10-min interval of observation (Table 3). There were also no significant differences in the dose of *M. oleifera* leaf tea in MF and IF (Table 4 and Table 5).

### 2.5. Testosterone Hormone and Relative Testis Weight

**Figure 4:**
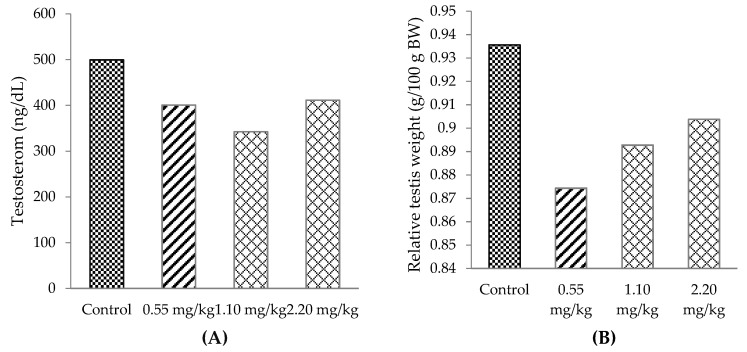
Testosterone hormone (**A**) and relative testis weight (**B**) of male rats administered different doses of *M. oleifera* leaf tea for 30 days (one-way ANOVA). There were no significant differences at *p* < 0.05. Data are mean values ± standard deviation (error bars).

There was no significant alteration of the testosterone level or relative testis weight in all treated groups when compared with the control group (Figure 4A and Figure 4B, respectively).

### 2.6. Histological of Testis 

**Figure 5:**
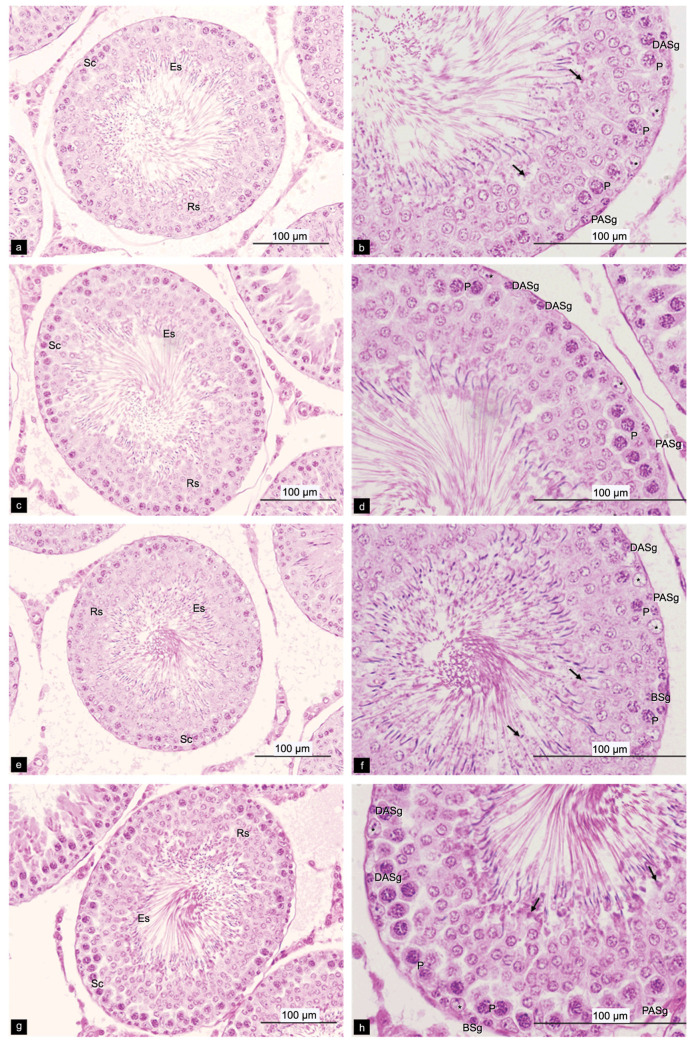
Photomicrographs of rat testis sections stained with H&E, showing the stages VII or VIII of the seminiferous epitheliums. (**a**,**b**) Control group; (**c**,**d**) Rats received *M. oleifera* leaf tea 0.55 mg/kg; (**e**,**f**) Rats received *M. oleifera* leaf tea 1.10 mg/kg; (**g**,**h**) Rats received *M. oleifera* leaf tea 2.20 mg/kg. The black arrow (→): Residual bodies; DASg: Dark type A spermatogonia; PASg: Pale type A spermatogonia; BSg: Type B spermatogonia; Sc: Spermatocytes; P: Pachytene primary spermatocytes; Rs: Round spermatids; Es: Elongate spermatids; *: Sertoli cells.

All the treatment groups had significantly increased seminiferous tubule diameter (Table 6), type A spermatogonia (*p* < 0.01) and spermatogonia efficiency (*p* < 0.05) in comparison with the control groups (Table 7 and Table 8). In addition, the rats treated with *M. oleifera* leaf tea at dosages of 1.10 and 2.20 mg/kg had a significantly increased number of Sertoli cells (*p* < 0.05) and the dose of 0.55 mg/kg increased the total spermatogenic cell (*p* < 0.01) compared to the control group (Table 7 and Table 8). The seminiferous epithelium cells are demonstrated in Figure 5.

## 3. Discussion

*M. oleifera* leaves are used in Thai recipes and traditional medicine. However, there is little scientific data regarding the use of this plant and its effects on the male reproductive system. Normally, *M. oleifera* leaves are consumed directly or brewed in near-boiling water. This method of producing *M. oleifera* leaf tea extract using hot water is familiar to Thai people who are accustomed to consuming this type of tea. It contains essential phytochemical compounds, especially total phenols and flavonoids. Phenolic compounds were easily eluded by polar solvent [27,28], as reported in the study on the *N*. *nucifera* petal extract [29]. In the present study, the phenolic compounds eluded in water were shown to be at a higher concentration than flavonoids. This is due to the polarity solvent that plays an important role in the phenolic and certain types of flavonoid solubility because flavonoids are also phenolic compounds [30]. 

An HPLC chromatogram of *M. oleifera* leaf tea presented ferulic acid, caffeic acid, chlorogenic acid, gallic acid, quercetin, and kaempferol. Similarly, the previous study found phenolic and flavonoid compounds in high concentrations in the *M. oleifera* leaves including compounds such as gallic acid, chlorogenic acid, ellagic acid, ferulic acid, caffeic acids, *p*-coumaric or ferulic, kaempferol, quercetin, and vanillin [31]. Phenolics and flavonoids have been reported to have a strong natural antioxidant properties [14], free radical scavenging ability and high potential to inhibit LPO, protein oxidation, AOPP, AGEs and DNA damage [27,32], leading to the prevention of cellular damage [24]. Furthermore, this study showed that the *M. oleifera* leaf tea was more effective for ABTS radical scavenging and H_2_O_2_ scavenging than gallic acid. These assays are based on a hydrogen donor for the radical scavenging or antioxidant reaction [33,34]. The *M. oleifera* leaf tea showed potential inhibition of LPO and AGEs formation. Lipids, proteins and carbohydrates are the macromolecules of the basic components in the living cell, which are the targets of free radicals [25]. Thus, this tea had rich total phenols and flavonoids resulting in strong antioxidant activity and inhibited the formation of LPO and AGEs.

The male rats treated with *M. oleifera* leaf tea at the dosages of 0.55, 1.10, and 2.20 mg/kg for 30 days significantly increased (*p* < 0.05) courtship behavior and in the first and second 10-min interval of observation compared with the control group during the entire 30-min observation period. This experiment demonstrated that the *M. oleifera* leaf tea could increase courtship behavior, which is an important pre-copulatory activity [35,36] resulting from sexual motivation. This is in contrast to the study on *K. Parviflora,* which was purported to be able to enhance sexual activity [37]. It is possible that the extraction factors and type of plants used played a role in making the results of this experiment different than anticipated [28,30]. Sexual behavior is controlled by a complex sequencing of motor patterns and multisensory stimulation [38]. Phenols and flavonoids act as antioxidants and might activate dopamine secretion as they operate in the hypothalamic region and medial amygdala leading to successful sexual behavior [38] in male rats treated with *M. oleifera* leaf tea.

The flavonoids have a chemical structure similar to that of cholesterol and other steroids which is characterized by the C6-C3-C6 group in which two benzene rings are connected by a three-carbon segment. Their core structures are important for a positive effect on testicular function and steroidogenesis, and they may influence androgen production in Leydig cells [39] and could activate androgen receptors of Sertoli cells. Therefore, flavonoids in *M. oleifera* leaf tea may have a role in Sertoli cells, increasing spermatogenesis [40], sperm quality [41] and sexual behavior [39]. In the brain, testosterone metabolizes to estradiol or dihydrotestosterone (DHT), which plays a role in the sensory stimulation from a receptive estrous female and facilitates the appetitive and consummatory sexual behavior [42]. The flavonoids act to provide multisensory stimulation and increase sex drive [42]. However, the testosterone level was not affected by *M. oleifera* leaf tea. It is possible that the tea may facilitate the sensory stimulation in male rats from receptive estrous females and might activate dopamine secretion leading to increased courtship behavior. 

Likewise, consumption of the white seed of *M. oleifera* by the male resulted in unchanged levels of testosterone, follicular stimulating hormone (FSH) and luteinizing hormone (LH) [43]. Testosterone hormone regulation is under the control of the hypothalamus through the work of gonadotropin-releasing hormone and is inhibited by testosterone via a negative feedback mechanism [44]. It might be possible that the serum testosterone was maintained at normal levels by the negative feedback mechanism and therefore did not alter after the *M. oleifera* leaf tea treatment.

*M. oleifera* leaf tea in all doses did not affect relative testis weight. The results of this study differed from a previous study in that testicular and epididymis weight increased after treatment with hexane *M. oleifera* extracted in male mice [45]. Type of solvent in extraction affected the amount of phytochemical contents, for example, *N*. *nucifera* petals extracted with hot water had higher phytochemical contents than 95% ethanol [29]. Therefore, the method of solvent extraction and the species of animal might have an effect on testicular weight in the animal model [45].

The male rats receiving *M. oleifera* leaf tea had significantly increased seminiferous tubule diameter, epithelium height, epithelium area, as well as an increase in type A spermatogonia and spermatogonia efficiency when compared with the control group. The rats treated with *M. oleifera* leaf tea at the doses of 0.55 and 1.10 mg/kg showed significant increases in the luminal area when compared with the control group. These results were similar to *B. rotunda* [35] and *M. oleifera* leaf extract with hexane [46], which increase seminiferous diameter due to increased spermatogenesis and result in the expansion of the seminiferous epithelium [35,41,45]. 

In addition, the *M. oleifera* leaves extract affected sperm characteristics along important parameters associated with male reproductive function in relation to fertility [45,46]. The study showed higher numbers of type A spermatogonia and spermatogonia efficiency which indicated that type A spermatogonia can divide by themselves to create a reserve of cells. For spermatogonia efficiency, the displayed values indicated the mitotic ability of type A spermatogonia differentiated to pachytene primary spermatocytes [47]. Consistently, *B. rotunda* juice at a dose of 120 mg/kg had significantly increased spermatogonia efficiency and was presumed to have a significant spermatogenesis effect on sperm production [48]. Because total phenols and flavonoids in *M. oleifera* leaf tea had scavenged free radicals, inhibited oxidative stress, stimulated type A spermatogonia proliferation, and differentiated to pachytene primary spermatocytes, this resulted in an increase of the total spermatogenic cells [49]. It implied an increase in seminiferous tubule diameter, epithelium area and luminal area. Moreover, the male rats that received 0.55 mg/kg in this study had the most effective increase in the seminiferous tubule diameter, epithelium area, luminal area, and total spermatogenic cell count. It is possible that a dose of 0.55 mg/kg of *M. oleifera* leaf tea may be appropriate for total phenols and flavonoids [4].

The present study found that, in mature rats treated with *M. oleifera* leaf tea at doses of 1.10 and 2.20 mg/kg, the number of Sertoli cells was significantly increased. The increase of Sertoli cells resulted in an increased number of germ cells. The germ cells secrete glial cell line-derived neurotrophic factor (GDNF), which stimulates type A dark spermatogonia to divide by themselves by way of mitotic division to create a reserve and differentiate to type A pale spermatogonia and then differentiate into type B spermatogonia [44]. This results in increased sperm concentration and sperm viability [50]. In addition, the natural antioxidants can have antioxidative stress effects. The effects on seminal plasma result in the increase of cell membrane permeability, leading to an improvement of sperm survivability [25], which could positively affect male reproduction [24,25]. Moreover, the previous study had reported that rats treated with *K. parviflora* extract had increased sperm concentration and demonstrated that the Sertoli cells had abundantly higher lysosome and prominent granules under the transmission electron microscope [40]. Thus, the quantity and quality of Sertoli cells affected spermatogonia and caused an increase in spermatogenesis.

The *M. oleifera* leaf tea had increased free radical scavenging and inhibited LPO and AGEs in the cell-free system, which implies that in an animal model there would likely be a reduction of oxidative stress, resulting in increased potential of the male reproductive parameters. In addition, *M. oleifera* leaf tea at the dose of 0.55 mg/kg showed the highest potential benefits for the reproductive function in male rats. Therefore, the antioxidative phytochemicals in *M. oleifera* leaf tea act as antioxidants, enhancing sexual function and the male reproduction system.

## 4. Materials and Methods

### 4.1. Chemicals and Reagents 

The chemicals and reagents, including formic acid and acetic acid (glacial), were purchased from Merck KGaA (Darmstadt, Germany). Potassium acetate, 2,2-diphenyl-1-Picrylhydrazyl (DPPH), 2,20 -Azino-di-(3-Ethylbenzthiazoline Sulfonic acid) (ABTS), Folin-Ciocalteu reagent, sodium acetate and thiobarbituric acid were purchased from Sigma-Aldrich (St. Louis, MO, USA). Ferulic acid, caffeic acid, chlorogenic acid, gallic acid, quercetin and kaempferol using for phytochemical standards were purchased from Sigma-Aldrich (St. Louis, MO, USA).

### 4.2. Plant Collection and Extraction

The completed leaves of *M. oleifera* were collected from the San Pu Loei subdistrict, Doi Saket district, Chiang Mai Province, Thailand (18°49′17.5″ N, 99°03′17.9″ E), during August 2018. The sample was deposited and authenticated at the Herbarium, Faculty of Pharmacy, Chiang Mai University, and the voucher number was 023240. The leaves were washed, steamed and dried at 60 °C. The dried leaves were pulverized and stored at 4 °C before use. The leaves were extracted one time with hot distilled water at 75–80 °C like tea for 5 min. The extract was filtered with a syringe filter and diluted with distilled water before experimentation.

### 4.3. Total Phenolic Contents

Total phenolic content in the *M. oleifera* leaf tea extract was determined by the Folin–Ciocalteu reagent and gallic acid was used as a standard [51]. A 50 µL of sample solution volume was added into a 5 mL tube containing 500 µL of 10% Folin–Ciocalteu reagent. Then, 500 µL of 1 M sodium carbonate (Na_2_CO_3_) was added and then incubated at room temperature for 15 min. The absorbance was measured at 765 nm by spectrophotometer (Shimadzu UV-2401PC, Thermo Fisher Scientific, Waltham, MA, USA). The total phenolic content was expressed in µg of gallic acid equivalents per mg plant dried weight.

### 4.4. Total Flavonoids Contents

Total flavonoids content was estimated by colorimetric assay and quercetin was used for calibration [51]. Of the sample, 100 µL was added to a 5 mL tube. Then, 50 µL of 10% aluminum chloride was added and incubated at room temperature for 30 min. Then, 50 µL potassium acetate and 700 µL of distilled water were added. The absorbance was measured at 415 nm by a spectrophotometer. The total flavonoid content was expressed in µg of quercetin equivalents per mg plant dried weight.

### 4.5. Total Alkaloid Contents

Total alkaloid contents were determined using the modified method of Van Tan [52] and 250 µL of the sample was added to test tube. Then, 1.25 mL of Phosphate Buffered Saline (PBS) pH 4.7 and 1.25 mL bromocresol green solution were added. Then, the solution was incubated at room temperature for 30 min and 2.5 mL of chloroform was added. The organic layer was measured at 470 nm by a spectrophotometer. The total flavonoid content was expressed in ng of morphine equivalents per mg plant dried weight.

### 4.6. Analysis of Phytochemical Content by High-Performance Liquid Chromatography (HPLC)

*M. oleifera* leaf tea was screened for phytochemical profiles with a high-performance liquid chromatography (HPLC)-diode array (Agilent 1260 Infinity Binary LC, Santa Clara, CA, USA) [53]. The HPLC condition was comprised of Purospher^®^ Star PR-18 endcapped column (150 × 4.60, 5 µm). The mobile phase consisted of 92% A (0.1% formic acid in water) and 8% B (acetonitrile), maintained for 10 min. Then B was increased to 14% in 24 min, to 23% in 35 min, to 24% in 60 min and the sample injection volume was 10 µL. The spectra were determined at 250 nm and 330 nm. The spectra between 200 to 400 nm were collected and identified. The chromatographic peak was achieved by comparing the retention times and spectral characteristics of the eluted peaks with the standards.

### 4.7. Antioxidant Properties

#### 4.7.1. DPPH Radical Scavenging Assay

The free radical scavenging ability of *M. oleifera* leaf tea was evaluated by the DPPH radical scavenging capacity assay [51]. Then, 100 µL of the various concentrations of *M. oleifera* leaf tea was added into 1 mL of 0.004% DPPH solution in methanol. The solution was incubated for 30 min in the dark. The absorbance was measured at 515 nm using a microplate reader (Bio Tek Synergy H4 Hybrid Microplate Reader, BioTek Instruments, Winooski, VT, USA) and gallic acid was used for positive control. The results were calculated and expressed as percentage of inhibition according to:

% inhibition = (A_DPPH_ − A_sample_)/A_DPPH_ × 100.

#### 4.7.2. ABTS Radical Scavenging Assay

The free radical scavenging activity of *M. oleifera* leaf tea was determined by ABTS radical cation decolorization assay [54]. ABTS was prepared by mixing 7 mM ABTS stock solution and 2.45 mM potassium persulfate (1:1). The mixture was stored and stood in the dark at 4 °C for 12–16 h before use. Then, the ABTS stock solution was diluted with distilled water to obtain an absorbance of 0.7 at 734 nm. Next, 200 µL of ABTS working solution was added to a 5 mL tube with 50 µL of the various concentrations of the leaf tea, followed by a 30 min incubation in darkness. The absorbance was measured at 743 nm using a microplate reader and gallic acid was used for positive control. The results were calculated and expressed as percentage of inhibition according to:

% inhibition = (A_ABTS_ − A_sample_)/A_ABTS_ × 100.

#### 4.7.3. Hydrogen Peroxide Scavenging Assay

The hydrogen peroxide (H_2_O_2_) scavenging activity of *M. oleifera* leaf tea was determined [55]. Five hundred microliters of the leaf tea were added to the 2.5 mL of 40 mM H_2_O_2_ solution in cooled water. Then, the solution was incubated for 10 min at room temperature. Before and after incubation, the solution absorbance was measured at 230 nm by a spectrophotometer. Gallic acid was used for positive control. The results were calculated and expressed as percentage of inhibition according to:

% inhibition = (A_H_2_O_2__ − A_sample_)/A_H_2_O_2__ × 100.

### 4.8. Lipid Peroxidation Assay

The inhibition of lipid peroxidation of *M*. *oleifera* leaf tea was determined by a thiobarbituric acid-reactive species (TBARS) assay [56]. This included linoleic acid emulsion (125 µL, 10 mM in 1M Phosphate Buffered Saline (PBS), pH: 7.4) and 12.5 µL of FeSO_4_ solution (0.07 M). Then, 50 µL of the leaf tea was added and mixed with the solution. Finally, the mixture volume was made up to 287.5 µL with distilled water and the solution was incubated for 30 min at room temperature and 225 µL of 0.85% normal saline solution, 500 µL 10% trichloroacetic Acid (TCA), and 100 µL of thiobarbituric acid (TBA) were added before boiling at 95°C for 30 min. The cool mixture was centrifuged at 3500 rpm for 10 min. The absorbance of the supernatant was measured at 532 nm by a microplate reader. Percent inhibition was calculated following the equation: positive

% Inhibition = (OD_control_ − OD_sample_)/OD_control_ × 100.

### 4.9. Inhibition of Advance Glycation End Products (AGEs) Formation

The anti-AGEs assay was evaluated according to a previous study [29]. Briefly, 50 µL of BSA (1 mg/mL in 0.2 M PBS, pH: 7.4) and 50 µL of D-glucose (1 M) were added to a 96 well-plate. The leaf tea solution was added and then incubated at 37 °C for 96 h in the incubator before AGEs measurement with a microplate reader. Excitation wavelength 360 nm and emission wavelength 460 nm were measured, and percent inhibition was calculated following the equation: positive

% Inhibition = (OD_control_ − OD_sample_)/OD_control_ × 100.

### 4.10. Animals

Thirty-two mature male *Wistar* rats aged 6–8 weeks and weighing 220–240 g were purchased from Nomura Siam International CO., LTD., (Bangkok, Thailand). The animals were housed under standard environmental conditions, controlled temperature at 25 ± 2 °C, 12 h dark/12 h light cycle and were a fed standard diet and filtered water ad libitum at the Animal Laboratory Building, Faculty of Medicine, Chiang Mai University. The animals were acclimatized in the housing condition for at least one week before studying. The experimental procedure was approved by the Animal Ethics Committee, Faculty of Medicine, Chiang Mai University (No.22/2018) and agreed with the institutional guides for the Animal Care and Use Laboratory animal.

### 4.11. Experimental Design

The male rats were randomly separated into four groups (n = 8 each), group I (control group) received distilled water (1 mL/day). The other groups were fed *M. oleifera* leaf tea at 0.55, 1.10 and 2.20 mg/kg, respectively for 30 days. Sexual behavior was tested during days 28–30 of the *M. oleifera* leaf tea application by using female rats. Finally, the rats were sacrificed and blood was collected by cardiac puncture. Testis were removed, trimmed of fat, and weighed before histological study.

### 4.12. Sexual Behavior Testing Procedure

Sexual behavior of the male rats was observed by using a rectangular cage under a dim red-light condition [35]. First, the rats were accommodated for 10 min before testing. Then, sexual behavior was recorded for 30 min after introducing female rats in the estrous phase [35,37,57,58,59,60]. Behavioral parameters including courtship, mount latency (ML), intromission latency (IL), mount frequency (MF), intromission frequency (IF), and copulatory efficiency (CE) were recorded throughout the whole period by video. Vaginal fluid of female rats were smeared and sperm was determined in order to confirm male ejaculation [37].

### 4.13. Testosterone Assay

Testosterone level in each blood sample was evaluated with the cooperation of the Chiang Mai Veterinary Laboratory Centre, Chiang Mai, Thailand.

### 4.14. Histological of Testis Evaluation

The right testis of each animal was fixed in Bouin’s fixative for paraffin work, and each section’s thickness was 4 μm. The slide was stained with hematoxylin and eosin (H&E) and digital micrographs were taken under a light microscope (Olympus AX70). The diameters, epithelium high, epithelium area, and luminal area in stages VII and VIII of the seminiferous epithelium cycle were measured [35,61,62]. Sertoli cells and spermatogenic cells were identified [48,63]. All parameters were investigated using the image J process version 1.5, analyzing program.

### 4.15. Statistical Analysis

The data were described with mean ± SE. The half-maximal inhibitory concentrations (IC50) of DPPH, ABTS, H_2_O_2_ scavenging, LPO, AOPP, and AGEs were calculated using Excel Microsoft 365 and were compared with the standard using the independent *t*-test. The mean of both dose and interval of courtship behavior, MF, IF were analyzed using a two-way analysis of variance (ANOVA), followed by one-way ANOVA and subsequently a least significant difference (LSD) multiple comparisons test. The mean of the testosterone hormone level, relative testis weight, luminal area, seminiferous epithelium cell, seminiferous epithelium cell ratio, courtship behavior, and ML in 30 min were analyzed by one-way ANOVA followed by LSD. The means of MF, IF, IL, CE in 30 min were calculated by the Kruskal–Wallis test. The seminiferous tubule diameter, epithelium high, and epithelium area were analyzed using the Kruskal–Wallis, followed by Mann–Whitney U test. SPSS 22.0 was employed for all statistical analyses. The significance level was set at *p* < 0.05.

## 5. Conclusions

In conclusion, *M. oleifera* leaf tea contained rich total phenols and flavonoids and had potential for antioxidant activities in the cell-free system. The *M. oleifera* leaf tea enhanced courtship behavior and reproductive functions. The specific mechanisms of bioactive compounds and the use of *M. oleifera* leaf tea in humans as a nutritional and male reproductive supplement should be further studied.

## Figures and Tables

**Table 1:** The half-maximal inhibitory concentration (IC50) values of DPPH, ABTS, H_2_O_2_ scavenging activity, LPO, AOPP, and AGEs of gallic acid and *M. oleifera* leaf tea.

Parameters	*M*. *oleifera* Leaf Tea (µg/mL)	Gallic Acid (µg/mL)
DPPH	19.35 ± 0.43 *	0.81 ± 0.00
ABTS	4.12 ± 0.15 *	28.42 ± 2.07
H_2_O_2_	3.59 ± 0.12 *	16.23 ± 0.16
LPO	37.82 ± 0.61 *	17.98 ± 0.12
AGEs	0.65 ± 0.00 *	25.51 ± 0.08

* Significant compared with gallic acid at *p* < 0.05 (independent *t*-test was performed).

**Table 2:** Sexual behavior parameters of male rats treated with *M. oleifera* leaf tea and the control group.

Parameters	Control (*n* = 8)	0.55 mg/kg (*n* = 8)	1.10 mg/kg (*n* = 8)	2.20 mg/kg (*n* = 8)
Courtship (×10 ^3^ s)	0.40 ± 0.07 ^a^	0.65 ± 0.06 ^b^	0.67 ± 0.09 ^b^	0.65 ± 0.07 ^b^
MF	28.23 ± 11.54	1.88 ± 1.22	17.25 ± 7.56	25.13 ± 11.18
IF	21.50 ± 10.50	0.13 ± 0.13	13.88 ± 7.72	19.75 ± 10.73
ML (×10 ^3^ s)	0.40 ± 0.02	1.03 ± 0.03	0.29 ± 0.02	0.31 ± 0.02
IL (×10 ^3^ s)	1.04 ± 0.03	1.58 ± 0.02	0.94 ± 0.03	0.59 ± 0.03
CE (IF/MF)	0.37 ± 0.15	0.01 ± 0.01	0.40 ± 0.16	0.40 ± 0.13

^a,b^ Different characters indicate significant differences between groups in the row at *p* < 0.05; one-way ANOVA followed by LSD in courtship behavior and ML, compared to the control group; MF, IF, IL and CE using the Kruskal-Wallis test. MF: mount frequency; IF: intromission frequency; ML: mount latency; IL: intromission latency; CE: copulatory efficiency.

**Table 3:** Courtship behavior of male rats treated with *M. oleifera* leaf tea and the control group observed in three 10-min intervals over the 30-min period.

10 min Interval Observation	Control (*n* = 8)	0.55 mg/kg (*n* = 8)	1.10 mg/kg (*n* = 8)	2.20 mg/kg (*n* = 8)
1st	208.25 ± 40.95 ^a^	325.00 ± 33.66 ^b^	340.75 ± 35.75 ^b^	351.25 ± 38.03 ^b^
2nd	109.75 ± 24.17 ^c^	195.00 ± 19.66 ^d^	196.50 ± 25.57 ^d^	182.88 ± 21.49 ^d^
3rd	85.88 ± 18.90 ^c^	133.13 ± 19.80 ^cd^	131.25 ± 39.69 ^cd^	120.50 ± 21.10 ^cd^

^a,b,c,d^ Different characters indicate the significant differences between the groups in the raw and between the intervals at *p* < 0.05 (two way ANOVA followed by one-way ANOVA and LSD, compared to the control group, and the Kruskal-Wallis test in the 3rd interval).

**Table 4:** Mount frequency of male rats treated with *M. oleifera* leaf tea and the control group observed in three 10 min interval over 30 min period.

10 min Interval Observation	Control (*n* = 8)	0.55 mg/kg (*n* = 8)	1.10 mg/kg (*n* = 8)	2.20 mg/kg (*n* = 8)
1st	11.50 ± 5.23	1.25 ± 0.84	10.38 ± 3.34	12.88 ± 5.39
2nd	8.50 ± 4.36	0.5 ± 0.38	4.63 ± 2.77	5.88 ± 3.25
3rd	8.13 ± 4.01	0.13 ± 0.13	2.25 ± 1.97	6.38 ± 2.95

There were no significant differences (two-way ANOVA).

**Table 5:** Intromission frequency of male rats treated with *M. oleifera* leaf tea and the control group observed in the first, second and third 10 min interval over 30 min period.

10 min Interval Observation	Control (*n* = 8)	0.55 mg/kg (*n* = 8)	1.10 mg/kg (*n* = 8)	2.20 mg/kg (*n* = 8)
1st	7.63 ± 4.39	0.13 ± 0.13	7.63 ± 3.70	9.13 ± 5.19
2nd	7.25 ± 4.20	0.50 ± 0.00	4.12 ± 2.71	5.50 ± 3.17
3rd	6.63 ± 3.82	0.00 ± 0.00	2.12 ± 1.82	5.13 ± 2.55

There were no significant differences (two-way ANOVA).

**Table 6:** Seminiferous tubule diameter, epithelium high, epithelium area, and luminal area at stage VII-VIII of male rats administered with different doses of *M. oleifera* leaf tea for 30 days.

Parameters	Control (*n* = 8)	0.55 mg/kg (*n* = 8)	1.10 mg/kg (*n* = 8)	2.20 mg/kg (*n* = 8)
Seminiferous tubule diameter (µm)	333.10 ± 2.78 ^a^	376.00 ± 3.80 ^b^	350.75 ± 3.85 ^c^	358.40 ± 3.27 ^c^
Epithelium high (µm)	59.25 ± 0.58 ^a^	64.31 ± 0.77 ^b^	64.16 ± 0.78 ^b^	62.82 ± 0.67 ^b^
Epithelium area (×10^3^ µm^2^)	40.75 ± 1.02 ^a^	54.50 ± 0.79 ^b^	50.02 ± 0.82 ^c^	49.94 ± 0.65 ^c^
Luminal area (×10^3^ µm^2^)	29.85 ± 0.78 ^a^	33.06 ± 0.79 ^b^	26.93 ± 0.68 ^c^	29.34 ± 0.71 ^a^

^a,b,c^ Different characters indicate significant differences between groups in raw data at *p* < 0.05; one-way ANOVA followed by LSD in luminal area, compared to the control group. Other parameters were used Kruskal-Wallis test followed by Mann-Whitney U test, compared to the control group.

**Table 7:** Numbers of spermatogenic and Sertoli cell nuclei in cross-section of seminiferous tubule at stage VII-VIII of the male rats treated with *M. oleifera* leaf tea in the different dose for 30 days, compared with control.

Parameters	Control (*n* = 8)	0.55 mg/kg (*n* = 8)	1.10 mg/kg (*n* = 8)	2.20 mg/kg (*n* = 8)
Type A spermatogonia (×10^3^ cells)	0.37 ± 0.02 ^a^	0.45 ± 0.01 ^b^	0.46 ± 0.02 ^b^	0.45 ± 0.02 ^b^
Pachytene primary spermatocytes (×10^3^ cells)	0.57 ± 0.02	0.61 ± 0.03	0.62 ± 0.02	0.61 ± 0.01
Round spermatids (×10^3^ cells)	1.52 ± 0.17	1.72 ± 0.04	1.68 ± 0.06	1.69 ± 0.06
Total number of spermatogenic cells (×10^3^ cells)	2.46 ± 0.09 ^a^	2.93 ± 0.14 ^b^	2.75 ± 0.10 ^ab^	2.74 ± 0.09 ^ab^
Sertoli cell nuclei (×10^3^ cells)	0.17 ± 0.01 ^a^	0.21 ± 0.01 ^ab^	0.23 ± 0.01 ^b^	0.22 ± 0.01 ^b^

^a,b^ Different characters indicate significant differences between the group in raw data at *p* < 0.05 (one-way ANOVA followed by LSD) compared to the control group.

**Table 8:** The spermatogonia efficiency, meiotic index, Sertoli efficiency, Sertoli capacity, the Sertoli cell index (SEI) and the spermatozoa–Sertoli index (SSEI) of male rats administered with different doses of *M. oleifera* leaf tea for 30 days.

Parameter	Control (*n* = 8)	0.55 mg/kg (*n* = 8)	1.10 mg/kg (*n* = 8)	2.20 mg/kg (*n* = 8)
Spermatogonia efficiency	1.53 ± 0.06 ^a^	1.36 ± 0.05 ^b^	1.32 ± 0.03 ^b^	1.38 ± 0.06 ^b^
Meiotic index	2.68 ± 0.08	2.86 ± 0.07	2.72 ± 0.05	2.78 ± 0.07
Sertoli efficiency	8.63 ± 0.56	8.33 ± 0.50	7.26 ± 0.38	7.93 ± 0.55
Sertoli capacity	14.00 ± 0.89	13.43 ± 0.81	11.86 ± 0.67	12.89 ± 0.90
SEI	6.97 ± 0.52	7.62 ± 0.42	8.62 ± 0.47	8.02 ± 0.47
SSEI (10^5^)	5.43 ± 0.09	6.30 ± 0.13	7.29 ± 0.12	8.25 ± 0.12

^a,b^ Different characters indicate significant differences between the group in raw data at *P* < 0.05 (one-way ANOVA followed by LSD) compared to the control group.

## Data Availability

The authors declare that the data supporting the findings of this study are available with in the article.
